# Analysis of Vaccination Rates and New COVID-19 Infections by US County, July-August 2021

**DOI:** 10.1001/jamanetworkopen.2021.47915

**Published:** 2022-02-10

**Authors:** Diego F. Cuadros, F. DeWolfe Miller, Susanne Awad, Philip Coule, Neil J. MacKinnon

**Affiliations:** 1 Department of Geography and Geographic Information Science, University of Cincinnati, Cincinnati, Ohio; 2 Department of Tropical Medicine and Medical Microbiology and Pharmacology, University of Hawaii, Honolulu; 3 Infectious Disease Epidemiology Group, Weill Cornell Medicine-Qatar, Cornell University, Doha, Qatar; 4 Department of Emergency Medicine, Medical College of Georgia, Augusta University, Augusta; 5 Department of Population Health Sciences, Medical College of Georgia, Augusta University, Augusta

## Abstract

This cross-sectional study analyzes the association between vaccination rates and COVID-19 incidence by county from July to August 2021.

## Introduction

There is substantial variation in the spatial distribution of COVID-19 vaccination rates in the United States. We conducted an ecological data visualization analysis to assess the association of the heterogeneous distribution of vaccination coverage with the dynamics of COVID-19 during the third wave of the pandemic in the US.

## Methods

Institutional review board approval and informed consent were not necessary for this cross-sectional study because all data were deidentified and publicly available (Common Rule 45 CFR §46). This study follows the Strengthening the Reporting of Observational Studies in Epidemiology (STROBE) reporting guideline. COVID-19 data at county level from July 1 to August 31, 2021, were obtained from Johns Hopkins University.^1^ Data for cumulative full vaccination rates in the total population at county level were obtained from the Centers for Disease Control and Prevention COVID data tracker for the contiguous US.^2^ We excluded Colorado, Georgia, Texas, Virginia, and West Virginia owing to incomplete or unreliable vaccination data. Counties were classified as rural or urban based on the 2013 National Center for Health Statistics classification scheme.^3,4^ Temporal changes in COVID-19 incidence (ie, new cases per 100 000 people in each time interval) were estimated by generating 4 time intervals of equal length (ie, July 1-15, July 16-31, August 1-15, and August 16-31). Cumulative vaccination rates for each time interval were estimated for the last day of each interval. We aggregated vaccination rates in 4 groups: less than 30%, 30% to less than 40%, 40% to 50%, and greater than 50% for visual data analysis. We created bivariate maps and scatterplots illustrating spatial associations between COVID-19 incidence and vaccination rates per county using ArcGIS Pro version 2.8 (Esri).

## Results

**Figure 1.**
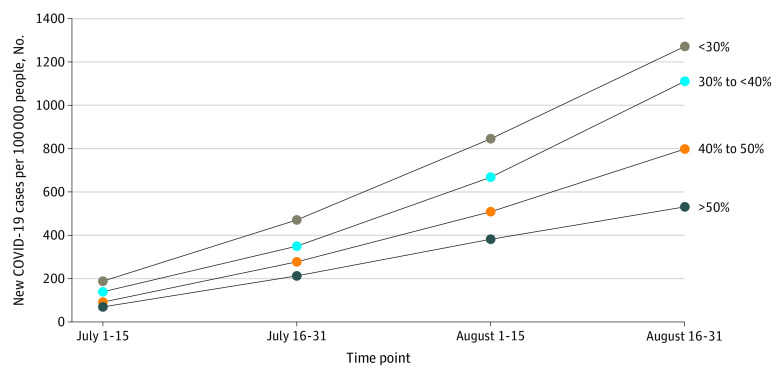
Association Between New COVID-19 Cases and Vaccination Rate by County Vaccination rates for each county were aggregated in 4 groups of less than 30%, 30% to less than 40%, 40% to 50%, and greater than 50%. Lines indicate temporal changes in new COVID-19 cases per 100 000 people during July 1 to 15, July 16 to 31, August 1 to 15, and August 16 to 31.

In areas with vaccination rates lower than 30%, COVID-19 infections per 100 000 people increased from 190 infections (95% CI, 188-193 infections) during July 1 to 15 to 1272 infections (95% CI, 1263-1280 infections) during August 16 to 31 (Figure 1). COVID-19 infections per 100 000 people in areas with vaccination rates higher than 50% increased from 71 infections (95% CI, 70-72 infections) during July 1 to 15 to 531 infections (95% CI, 529-532 infections) during August 16 to 31, and areas with lower vaccination rates (ie, <30%) had 2.4 more new COVID-19 infections per 100 000 people compared with areas with high vaccination rates (ie, >50%) during August 16 to 31. Rural counties accounted for 369 of 449 areas with lower vaccination rates (82.2%). Conversely, rural counties accounted for 131 of 376 areas with high vaccination rates (34.8%).

**Figure 2.**
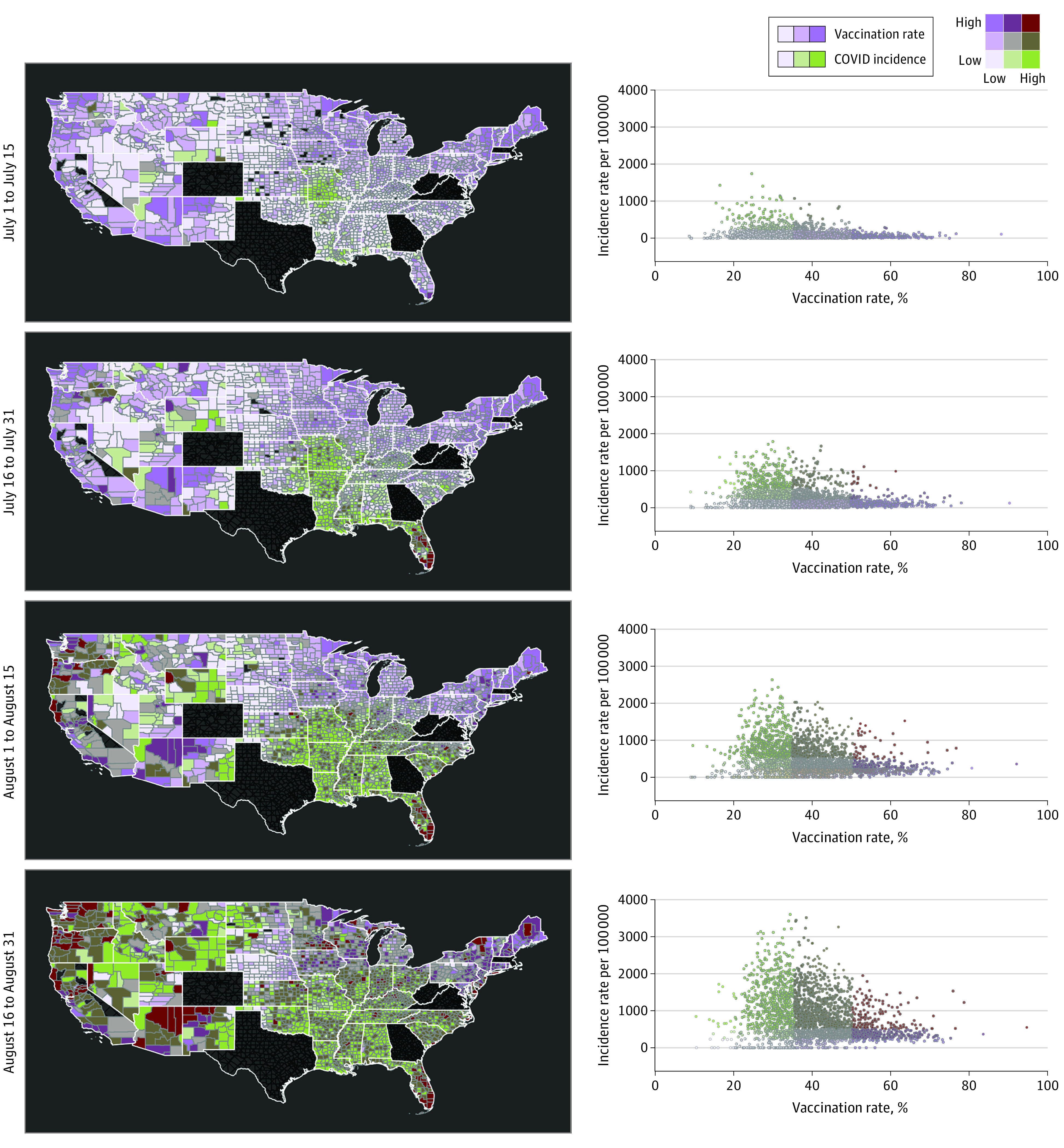
Bivariate Maps of the Association Between Vaccination Rate and New COVID-19 Cases by County Bivariate maps illustrate the spatial changes in the association between vaccination rates and new COVID-19 cases per 100 000 people (incidence rate) at the county level during July 1 to 15, July 16 to 31, August 1 to 15, and August 16 to 31 in 2021. Scatterplots indicate the association between these variables in each county; purple graduate colors, low (<30%), medium (30%-50%), and high (>50%) vaccination rates; green graduate colors, low (<250), medium (250-500), and high (<500) new COVID-19 cases per 100 000 people; dark green, counties with low vaccination rates and high COVID-19 incidence rates; dark purple, counties with high vaccination rates and low COVID-19 incidence rates; dark brown, counties with high vaccination rates and high COVID-19 incidence rates.

Bivariate maps in Figure 2 illustrate the spatial changes of COVID-19 incidence with respect to vaccination per county in the 4 time periods. Scatterplots show that while the epidemic emerged in areas with high vaccination (dark brown dots), COVID-19 incidence rates were decreased compared with low vaccination coverage areas, with increased dispersion of dots illustrating an increased number of new COVID-19 infections, shown as green dots. Western and northern parts of the country, along with Florida, were areas with increased percentages of vaccination rates (areas in purple in map for July 1-15). An increased number of new COVID-19 cases emerged in July 1 to 15 in Missouri and Louisiana, 2 states with decreased percentage of vaccination (areas in green in July 1-15). The epidemic grew in most states in the southern part of the US, including Arkansas, Oklahoma, Mississippi, Tennessee, South Carolina, and Florida, in July 16 to 31 to August 1 to 15. The epidemic intensified in these areas by August 16 to 31 but also grew in the western US.

## Discussion

In this cross-sectional study, we found a negative ecological association between vaccination rates and the surge of COVID-19 infections. Areas with low vaccination experienced a more intense surge of new cases during the third wave of the pandemic in the US, primarily driven by the Delta variant. The results presented here illustrate the association of the spatial heterogeneity of vaccination coverage with the overall COVID-19 epidemic in the country. Most counties with low vaccination rates (ie, <30%) were rural (82.2%). Rural areas in the US face many challenges in responding to the pandemic, including lower health care resources, compared with urban communities. These areas have been characterized by vaccination hesitancy, limited vaccine availability, and hospital staff shortages that can be associated with the successful distribution of vaccines and hence the vaccination campaign’s overall outcome.^5^

Our study had several limitations. These include that an ecological study like this is an approach for examining the association between factors and diseases by conducting analysis at the population level in specific areas; therefore, in ecological studies, owing to the lack of individual data, it is difficult to adjust for all potential confounding factors. Despite this limitation, our study found heterogeneous variation of COVID-19 incidence during the third wave of the pandemic, potentially associated with differential vaccination coverage. These findings suggest that successful containment of the epidemic may be achieved if vaccination rates are substantially increased to diminish the spatial heterogeneity of vaccination in the country and boost epidemic response in rural areas.
